# Identification of Tumor- and Immunosuppression-Driven Glioblastoma Subtypes Characterized by Clinical Prognosis and Therapeutic Targets

**DOI:** 10.3390/cimb48010103

**Published:** 2026-01-19

**Authors:** Pei Zhang, Dan Liu, Xiaoyu Liu, Shuai Fan, Yuxin Chen, Tonghui Yu, Lei Dong

**Affiliations:** 1 State Key Laboratory of Hearing and Balance Science and Key Laboratory of Molecular Medicine and Biological Diagnosis and Treatment (Ministry of Industry and Information Technology), School of Life Science, Beijing Institute of Technology, Beijing 100081, China; 17601052235@163.com (P.Z.); uanea548326@163.com (D.L.); sfan_bio@163.com (S.F.); cyx1036894604@163.com (Y.C.); 2 School of Economics, Jinan University, Guangzhou 510632, China; xyliu0075@jnu.edu.cn; 3 International Center for Interdisciplinary Statistics, School of Mathematics, Harbin Institute of Technology, Harbin 150001, China; 4 School of Physical and Mathematical Sciences, Nanyang Technological University, Singapore 637371, Singapore

**Keywords:** pathway-based signature, prognosis, immune infiltration, inherent driving, WGCNA, neural network model, Cox nomogram, therapeutic targets

## Abstract

Glioblastoma multiforme (GBM) is the most aggressive primary brain cancer (with a median survival time of 14.5 months), characterized by heterogeneity. Identifying prognostic molecular subtypes could provide a deeper exposition of GBM biology with potential therapeutic implications. In this study, we classified GBM into two prognostic subtypes, C1-GBM (*n* = 57; OS: 313 days) and C2-GBM (*n* = 109; OS: 452 days), using pathway-based signatures derived from RNA-seq data. Unsupervised consensus clustering revealed that only binary classification (cluster number, CN = 2; mean cluster consensus score = 0.84) demonstrated statistically prognostic differences. We characterized C1 and C2 based on oncogenic pathway and immune signatures. Specifically, C1-GBM was categorized as an immune-infiltrated “hot” tumor, with high infiltration of immune cells, particularly macrophages and CD4^+^ T cells, while C2-GBM as an “inherent driving” subtype, showing elevated activity in G2/M checkpoint genes. To predict the C1 or C2 classification and explore therapeutic interventions, we developed a neural network model. By using Weighted Correlation Network Analysis (WGCNA), we obtained the gene co-expression module based on both gene expression pattern and distribution among patients in TCGA dataset (*n* = 166) and identified nine hub genes as potentially prognostic biomarkers for the neural network. The model showed strong accuracy in predicting C1/C2 classification and prognosis, validated by the external CGGA-GBM dataset (*n* = 85). Based on the classification of the BP neural network model, we constructed a Cox nomogram prognostic prediction model for the TCGA-GBM dataset. We predicted potential therapeutic small molecular drugs by targeting subtype-specific oncogenic pathways and validated drug sensitivity (C1-GBM: Methotrexate and Cisplatin; C2-GBM: Cytarabine) by assessing IC_50_ values against GBM cell lines (divided into C1/C2 subtypes based on the nine hub genes) from the Genomics of Drug Sensitivity in Cancer database. This study introduces a pathway-based prognostic molecular classification of GBM with “hot” (C1-GBM) and “inherent driving” (C2-GBM) tumor subtypes, providing a prediction model based on hub biomarkers and potential therapeutic targets for treatments.

## 1. Introduction

Glioblastoma multiforme (GBM) is the most common and aggressive brain tumor. It exhibits severe intra- and inter-tumor heterogeneity, with a median survival time of only 14.5 months [1,2,3]. Multi-omics studies conducted by the Cancer Genome Atlas (TCGA) and the Chinese Glioma Genome Atlas (CGGA) have revealed complex genetic characteristics in GBM, such as co-deletions of chromosomes 1p and 19q, mutations in genes like IDH, PTEN, TP53, TERT, and ATRX, amplifications of EGFR, and disruptions in P53, Rb, and PI3K signaling pathways [4,5,6]. Classifying GBM based on molecular features enhances traditional pathology-driven diagnostics [6,7], enabling the identification of the molecular markers for disease diagnosis and patient stratification. The classification of GBM based on these abnormal molecules has gradually reduced the time from diagnosis to treatment, leading to a significant improvement in accuracy and specificity. However, challenges remain regarding the reliance on independent features and prognostic factors.

**Previous molecular subtypes of GBM still have limitations when it comes to improving patient prognosis.** The World Health Organization updated its guidelines to reclassify several brain tumor entities, particularly gliomas, considering both morphological and genetic variations and provided the isocitrate dehydrogenase (IDH, an enzyme that catalyzes the conversion of isocitrate to alpha-ketoglutarate in the citric acid cycle in brain tumor) gene mutation subtype [8]. Patients with IDH1 mutations, which occur in about 5% of primary GBM patients cases, have a better prognosis compared to those with IDH1 wildtypes [9]. However, despite this advantage in prognosis, IDH1 mutation in GBM patients is limited. Verhaak et al. proposed four GeneExp subtypes based on gene expression—proneural, neural, classical, and mesenchymal—defined by aberrations of EGFR, NF1, and PDGFRA/IDH1, providing a unified framework for transcriptomic and genomic stratification of GBM [7]. Wang et al. identified the neural subtype originating from non-tumor cells within the tumor microenvironment [10,11]. They also included methylation status to classify GBM into six methylation subtypes (clusters M1 to M6), with cluster M5 identified as the G-CIMP subtype [6]. This classification complements the analysis of gene mutation and transcription, further subdividing the proneural subtype into G-CIMP-positive and -negative groups. DNA methylation status in the MGMT promoter region can predict response to temozolomide treatment [12], but this is applicable only to the non-recurrent classical subtype of GBM. However, GeneExp subtypes and methylation subtypes lack clear prognostic differences and exhibit overlaps among subgroups. Recently, Park J. et al. identified three new GBM subtypes—mitotic (characterized by association with the methylated MGMT gene promoter and sensitive to TMZ treatment), intermediate, and invasive subtypes—through the analysis of four large-scale gene expression profiles [13]. Wang et al. extended the integration of MS-based proteomics to identify GBM subtypes differing in infiltrating macrophages and the distribution of specific immune cell types [14], and Ravi et al. employed spatially resolved multi-omics in glioblastoma samples and identified segregated niches hallmarked by immunological and metabolic stress factors [15], highlighting the need to consider the state of the tumor microenvironment (TME) for optimizing tumor classification.

Pathway-based analysis of transcriptomic cancer data, which captures biological interaction among genes, offers a more powerful and robust approach for molecular classification than traditional gene-based analysis. Pathway-based analysis evaluates a predefined aggregation of genes within functional pathways [16]. This approach utilizes information from multiple loci within a functional signaling pathway, leading to more stable and robust results compared to single gene analyses [17,18]. In the context of GBM, which is highly heterogeneous and complex, identifying distinct prognostic subtypes is essential for improving patient outcomes. Garofano et al. [17] demonstrated the effectiveness of a pathway-based classification method by analyzing single-cell and bulk RNA sequencing data. They found that the mitochondrial subtype reliant on oxidative phosphorylation is associated with better clinical outcomes, whereas the glycolytic/plurimetabolic subtype relies on aerobic glycolysis, amino acid, and lipid metabolism [17]. Our study aims to utilize pathway-based gene expression data to achieve molecular classification of GBM. By focusing on the collective behavior of gene sets within functional pathways, pathway-based classification offers a more robust and biologically meaningful analysis. This method inherently achieves dimension reduction by analyzing gene sets rather than individual genes [19,20], thus addressing the heterogeneity and complexity of GBM more effectively than traditional single-gene approaches.

In this study, we utilized pathway-based gene expression data and performed unsupervised clustering to identify two distinct prognostic GBM subtypes: cluster 1 (C1-GBM), characterized by an enriched immune microenvironment and active PD1 and MHC pathways (immune-infiltrated “hot” tumors), and cluster 2 (C2-GBM), defined by activation of cell cycle and G2/M checkpoint (inherent driving). We developed a neural network classifier to predict the C1 and C2 subtypes and validated it using the external dataset from CGGA-GBM. Subsequently, we constructed a Cox proportional hazards model integrating predictions from the neural network to generate a nomogram for prognostic survival prediction in the TCGA-GBM cohort. Using the Genomics of Drug Sensitivity in Cancer (GDSC) database, we evaluated the sensitivity of GBM cell lines to subtype-specific gene targets. Our findings identified immune and inherent driving GBM subtypes via molecular signatures, enhancing prognostic predictions and informing the selection of targeted therapies for GBM patients.

## 2. Materials and Methods

### 2.1. Data Source

Gene expression profiles and clinical information regarding overall survival for the TCGA-GBM (*n* =166) were downloaded from https://xenabrowser.net/datapages/ (accessed on 14 January 2026) for use as the training set. An independently validated dataset from the Chinese Glioma Genome Atlas (CGGA) project was downloaded from http://www.cgga.org.cn/download.jsp (ID WESeq_325, accessed on 14 January 2026). The drug-related gene expression datasets GSE135222 and GSE78220 from the GEO dataset were downloaded from the Cancer Treatment Response gene signature DataBase (CTR-DB, http://ctrdb.cloudna.cn/home, accessed on 14 January 2026) for drug response analysis.

### 2.2. The Molecular Signatures Database (MSigDB) and Gene Set Variation Analysis (GSVA)

MSigDB is the largest collection of dataset for molecular markers [21]. It includes various gene expression features such as positional gene sets, strategy gene sets, regulatory target gene sets, computational gene sets, immune marker gene sets, feature gene sets, cancer marker gene sets, ontology gene sets, and cell type marker gene sets [21,22]. The GSVA package in R was used to compute sample-wise enrichment scores for MSigDB sets. Specifically, GSVA summarizes gene-level expression profiles into gene set-level activity scores, thereby transforming the original gene-by-sample expression matrix into a gene set-by-sample enrichment matrix. In this study, all MSigDB gene sets were jointly evaluated to generate an initial feature matrix comprising 35,206 gene set signatures across 166 TCGA-GBM samples. GSVA was implemented using the argument kcdf = “Gaussian”, with all remaining parameters set to their default values [23].

### 2.3. Unsupervised Consensus Clustering

Consensus clustering is a technique used to identify cluster members, allowing the differentiation of samples into subtypes based on various histological datasets. We utilized the ConsensusClusterPlus (v1.46.0) R package to perform clustering on the MSigDB gene set matrix (Row: pathway gene sets, *n* = 35,206; Colum: samples, *n* = 166) [24]. The clustering method employed was partitioning around medoids (PAM) with Minkowski distance and resampling 80% of the patients for 10 repetitions [25]. The cluster numbers (CNs) derived from this process were used for consensus assessment and survival analysis. The TCGA-GBM patients (*n* = 166) were divided into two clusters: C1-GBM (*n* = 57) and C2-GBM (*n* = 109).

### 2.4. Silhouette and Principal Component Analysis

The silhouette method is used to evaluate the quality of clustering in unsupervised machine learning algorithms [26]. It measures how well data points within a cluster are separated from those in other clusters. The silhouette score ranges from −1 to 1, with higher values indicating better-defined clusters, and the average silhouette width provides an overall assessment of clustering effectiveness [27]. Principal component analysis (PCA) is a statistical technique used for dimensionality reduction and feature extraction in data analysis and machine learning [28]. It aims to transform a dataset with potentially correlated variables into a new set of uncorrelated variables called principal components. PCA simplifies visualization by projecting high-dimensional data into lower dimensions, enabling easier assessment of clustering and data separation.

### 2.5. Mutation Analysis

To compare the C1-GBM and C2-GBM subtypes, we analyzed patient mutation data using the maftools R package [29]. Specifically, the CoOncoplot function depicted the mutation profiles of both subtypes, illustrating frequencies, co-occurrences, and exclusivities. For a more detailed statistical comparison, we employed the ForestPlot function to generate a forest plot, which revealed significant differences in gene mutations between the two types. This included effect sizes and confidence intervals, thus identifying genes significantly mutated in one subtype compared to the other.

### 2.6. Weighted Gene Co-Expression Network Analysis (WGCNA)

WGCNA was performed to explore gene–gene interaction patterns within the expression profiles of 964 prognostic tumor-related differentially expressed genes (DEGs) from the TCGA-GBM cohort [30,31]. First, we constructed a pairwise correlation matrix using Pearson correlation coefficients, with the similarity between genes i and j defined as $s_{ij}=corr(x_{i},x_{j})$, where $x_{i}$ and $x_{j}$ denote the expression profiles of genes i and j, respectively. The similarity matrix was then transformed into an adjacency matrix using a soft-thresholding power of β = 5: $a_{ij}=\;{|s_{ij}|}^{\beta }$, which increases the emphasis on stronger correlations. Next, a topological overlap matrix (TOM) was computed to capture both direct and indirect interactions based on shared neighbors. Hierarchical clustering was performed on the TOM-based dissimilarity to generate a gene dendrogram, and modules were identified using a minimum module size of 20 and a sensitivity of 4. Using a distance cutoff of 0.2, co-expression modules were defined; genes assigned to the grey module (unassigned genes) were excluded from downstream analyses. Hub genes were then identified from the non-grey modules based on high intramodular connectivity and strong module membership (∣MM∣>0.8); in total, nine hub genes were retained as candidate predictive biomarkers. Finally, module eigengene correlation heatmaps were generated to assess relationships among modules and to evaluate potential substructures.

### 2.7. Backpropagation (BP) Neural Network

The backpropagation (BP) neural network is a multilayer feedforward network trained using the backpropagation algorithm to minimize prediction error. We implemented a BP neural network in R using the NeuralNet package (v1.44.2) and used the expression levels of the nine hub genes as input features [32]. In the hidden layer, neuron activations were computed as h=f(Wx+b), where f(⋅) denotes the activation function, W is the weight matrix, x is the input vector (gene-expression features), and b is the bias vector. To constrain activations to the range [0, 1], we applied the sigmoid function $\sigma (z)=1/(1+e^{-z})$. The output layer activation was computed using a linear function $\hat{y}=W_{\mathrm{out}}h+b_{\mathrm{out}}$. Model performance was evaluated using repeated five-fold cross-validation: in each repeat, samples were randomly split into five folds, with four folds used for training and one fold held out for testing. This procedure was repeated 100 times (100 repeats), and accuracy, sensitivity, precision, and specificity were averaged across all folds and repeats. For external validation, the trained model was further assessed on the CGGA-GBM dataset (n=85), where subtypes were predicted using the BP-based classifier.

### 2.8. Differential Gene and Functional Analysis

Differential expression genes (DEGs) between C1-GBM and C2-GBM were obtained from the limma R package using the following screening criteria: genes with *p* value < 0.05 and fold change (FC) > |1.5| were considered to be statistically significant [33].

1. DEGs from Tumor vs. normal: The RNAseq of GBM (*n* = 166) and normal tissue (*n* = 5, from TCGA-GBM dataset) resulted in 13,618 DEGs.
2. DEGs between C1-GBM and C2-GBM clusters: The RNAseq data of C1-GBM (*n* = 57) and C2-GBM (*n* = 109) from GBM resulted in DEGs.
3. We selected the overlapping genes between DEGs from the C1-GBM vs. C2-GBM clusters and DEGs from tumor vs. normal. We obtained tumor-related DEGs that are significantly different between C1 and C2-GBM (tumor-related DEGs).
4. The tumor-related DEGs were used for the enrichment analysis of signaling pathways and Gene Ontology (R package clusterProfiler, false discovery rate (FDR) < 0.05 as statistically significant) [34].
5. The univariate-Cox was analyzed by integrating OS.time and patient status phenotype dataset (*n* = 166) and 964 prognosis tumor-related DEGs were obtained.

### 2.9. Tumor Microenvironment (TME) Analysis

We analyzed the tumor microenvironment using RNA sequencing data from the TCGA-GBM cohort (*n* = 166) with the xCell [35], quanTISeq [36], Immunophenoscore [37], and CIBERSORT [38]. The xCell, quanTISeq, and CIBERSORT algorithms utilize a database of gene expression signatures from known cell types to obtain the abundance of different cells in the sample. By comparing the gene expression patterns from our samples with these reference datasets, we could determine the relative presence of various cell types within the sample. The xCell, quanTISeq, and CIBERSORT were used for the unsupervised clustering of immune cells, resulting in two classifications of immune enrichment: “hot” tumors (highly enriched in immune cells) and “cold” tumors (low immune infiltration).

### 2.10. Drug Sensitivity Prediction and Verification

We explored the Genomics of Drug Sensitivity in Cancer database to identify target drug for GBM cell lines, specifically focusing on the subtype-specific target within the PD1 pathway and G2/M checkpoint. Subtype-specific targets from the top DEGs are analyzed for protein–drug interactions (using the DrugBank database, Version 5.0) and protein–chemical interactions (using the Comparative Toxicogenomics Database). This analysis identified small molecular drugs, which we then validated for effectiveness through Genomics of Drug Sensitivity in Cancer (GDSC). The data of cancer cell lines from GDSC were analyzed using the GSCA (https://guolab.wchscu.cn/GSCA/#/drug, accessed on 14 January 2026) to determine the relationship between drug sensitivity and gene expression.

### 2.11. Survival Model Construction

In the C1-GBM subgroup, univariable Cox regression was performed to evaluate associations between overall survival (OS) and the log_2_(FPKM + 1)-transformed expression levels of IL6 pathway-related genes. Genes with *p* < 0.05 were considered candidate variables, and the top five genes with the smallest *p*-values were selected for downstream modeling. Similarly, in the C2-GBM subgroup, univariable Cox regression was conducted to screen G2/M checkpoint pathway-related genes, using the same criteria (*p* < 0.05) and selecting the top five genes with the smallest *p*-values. In total, 12 variables from the TCGA-GBM cohort were included in the final model: age at diagnosis, gender, and the log_2_(FPKM + 1)-transformed expression levels of OSMR, STAT3, MYD88, IL6ST, and SOCS3 for C1-GBM, as well as ABRAXAS1, UBE2V2, PSMF1, PSMA8, and KAT5 for C2-GBM. Optimal cut-off points for risk stratification were determined using the maxstat (v0.7-25) R package. Model discrimination was assessed by time-dependent ROC analysis at 1- and 3-year time points using timeROC (v0.4).

### 2.12. Statistical Analysis

All statistical calculations were performed using the R software, version 4.3.2. The significance test of the regression coefficients was conducted using the two-sided Student’s *t*-test. Pearson’s correlation analysis was employed to compute correlation coefficients between factors. For prognostic analysis, survival curves were generated using the Kaplan–Meier method, with significance evaluated via the log rank test.

## 3. Results

### 3.1. Identification of Two GBM Prognostic Subtypes Through Unsupervised Clustering

**Figure 1:**
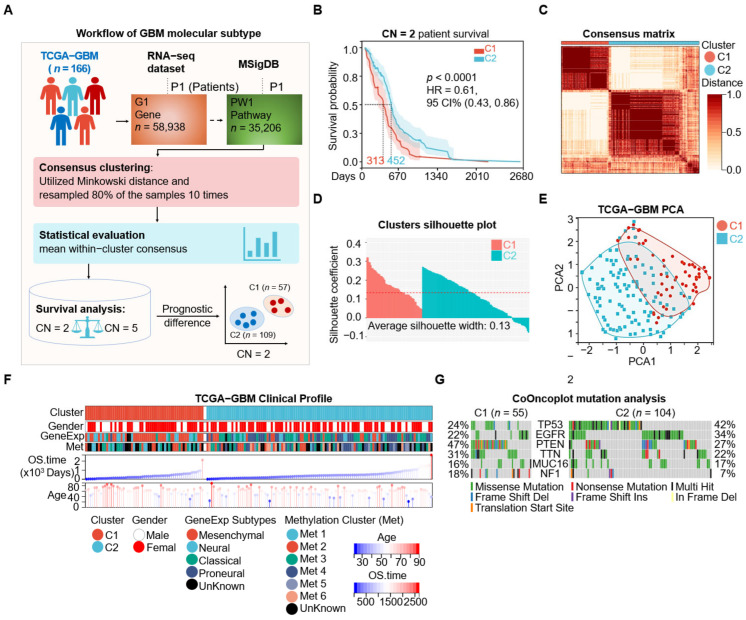
Identification of two GBM prognostic subtypes through unsupervised clustering. (**A**) Workflow of GBM molecular subtype. The figure outlines the comprehensive workflow for molecular subtyping of glioblastoma multiforme (GBM) using data from the TCGA-GBM cohort, which includes 166 patients. The analysis begins with gene expression data (G1 Gene, *n* = 58,938) from RNA sequencing (RNA-seq) datasets and integrates pathway data (PW1 Pathway, *n* = 35,206) from the Molecular Signatures Database (MSigDB). Consensus clustering was performed utilizing the Minkowski distance metric, resampling 80% of the samples ten times, and evaluating statistical significance by calculating mean cluster consensus. Survival analysis of the identified clusters revealed two clusters, C1 (*n* = 57) and C2 (*n* = 109), with significant differences in patient prognosis. (**B**) Kaplan–Meier survival curves for two clusters (CN = 2). The survival curves for cluster C1 (red) and cluster C2 (blue) show significant differences in survival probability (*p* < 0.0001, HR = 0.61, log rank test). The shaded areas indicate the 95% confidence intervals. The dashed lines indicate the median survival time for each cluster. (**C**) The consensus clustering matrix for CN = 2. The *x*-axis and *y*-axis represent individual patients, with the color intensity represents the correlation distance; darker shades indicate higher correlation and lighter shades indicate lower correlation. (**D**) Cluster silhouette evaluation (C1 in red and C2 in blue). The y-axis represents the silhouette coefficient, measuring an object’s similarity to its own cluster compared to others. The *x*-axis shows individual samples ordered within each cluster. Average silhouette width is 0.13, indicated by the dashed red line. (**E**) Principal component analysis (PCA) plot of GBM. The *x*-axis (PCA1) and y-axis (PCA2) represent the first and second principal components, respectively. (**F**) Clinical profile of TCGA-GBM samples, displaying clusters (C1 and C2), gender (male and female), gene expression subtypes (mesenchymal, neural, classical, proneural, unknown), and methylation clusters (Met 1-6, unknown). Heatmaps show the distribution of these features across samples, with the middle panel depicting overall survival (OS) time in days and age at diagnosis for each sample. Color bars represent clusters, gender, gene expression subtypes, methylation clusters, age, and OS time. (**G**) CoOncoplot of mutation profiles for top six mutated genes (TP53, EGFR, PTEN, TTN, MUC16, and NF1) in clusters C1 (*n* = 55) and C2 (*n* = 104). The y-axis lists the mutation frequency percentages for each gene within each cluster.

We utilized molecular signature sets from MSigDB via GSVA to form a comprehensive tumor process set of TCGA-GBM, comprising 35,206 signatures across 166 samples (Figure 1A). Unsupervised consensus clustering was then performed using the ConsensusClusterPlus R package with the number of clusters (CN) ranging from 2 to 10 to identify potential subtypes (Figure 1B). The evaluation of clustering consistency revealed that five clusters (CN = 5) reached the highest average cluster consensus score of 0.86, while two clusters (CN = 2) followed with an average score of 0.84 (Supplementary Figure S1A). Notably, increase in the area under the cumulative distribution function (CDF) was not substantial at CN = 5 (Supplementary Figure S1B). Moreover, survival analyses suggested limited separation among the five-cluster solution, with only two of the ten pairwise comparisons reaching statistical significance (Supplementary Figure S1C). In contrast, the two-cluster solution displayed a pronounced survival difference (*p* < 0.0001, log rank test; Figure 1B). Therefore, we selected CN = 2 for downstream analyses, defining two subtypes: C1-GBM (*n* = 57) and C2-GBM (*n* = 109). The consensus matrix for CN = 2 demonstrated stable cluster membership (Figure 1C). In addition, silhouette analysis (average silhouette width = 0.13) and principal component analysis (PCA) supported the separation between C1-GBM and C2-GBM (Figure 1D,E).

We next examined the clinical and molecular characteristics of these subtypes and compared them with previously reported GeneExp-Subtypes and Methylation Clusters [6,7]. C1-GBM showed a shorter overall survival (OS) time (313 days) and predominantly corresponded to the mesenchymal GeneExp subtype (*n* = 35, 61%) and methylation cluster Met2 (*n* = 13, 23%) among the six methylation clusters. Meanwhile, 2-GBM exhibited a longer OS time (452 days), was enriched for classical (34%) and proneural (34%) GeneExp subtypes, and aligned with methylation cluster Met4 (Figure 1F and Table 1). Mutation profiling revealed distinct patterns between the two subtypes. In C1-GBM, the most frequently mutated genes were PTEN (47%), TTN (31%), TP53 (24%), EGFR (22%), NF1 (18%), and MUC16 (16%). In C2-GBM, the highest mutation frequencies were observed in TP53 (42%), EGFR (34%), PTEN (27%), TTN (22%), MUC16 (17%), and NF1 (7%) (Figure 1G). Forest plot analysis further indicated a higher odds ratio of PTEN and NF1 mutations in C1-GBM, whereas TP53 mutation were more prevalent in C2-GBM; ATRX mutations were detected only in C2 (*p* < 0.05) (Supplementary Figure S1D). Collectively, these findings suggest that C1-GBM is more consistent with a mesenchymal-like molecular profile characterized by PTEN/NF1 alterations, while C2-GBM is more consistent with a proneural/classical-like profile with frequent TP53 mutations.

### 3.2. Identification of Featured Biomarkers for C1/C2 Subtypes via WGCNA

**Figure 2:**
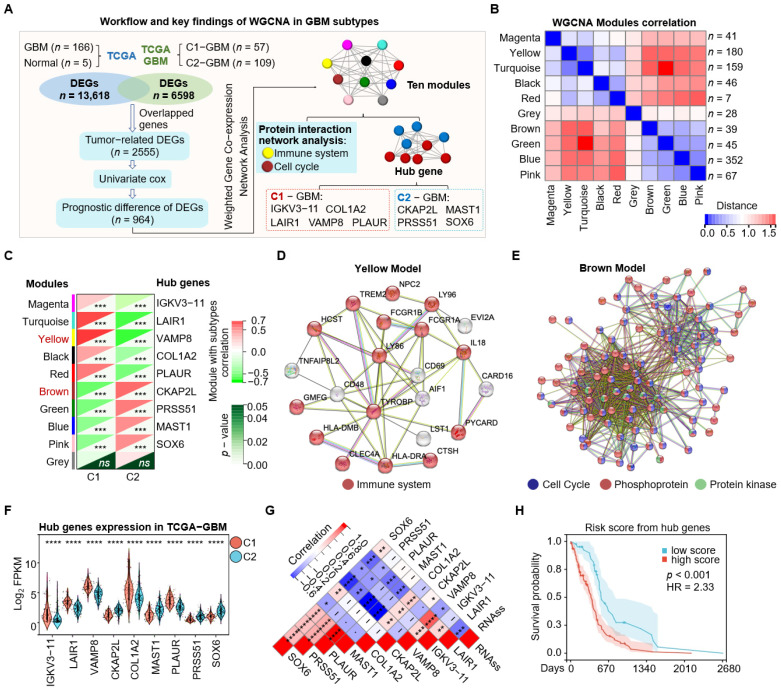
Identification of featured biomarkers for C1/C2 subtypes via Weighted Correlation Network Analysis (WGCNA). (**A**) Workflow and key findings of WGCNA in GBM subtypes. The analysis started with a cohort of GBM patients (*n* = 166) and normal controls (*n* = 5) from the TCGA database. Differentially expressed genes (DEGs) were identified: 13,618 DEGs from the comparison between GBM and normal tissues, and 6598 DEGs from the comparison between C1-GBM (*n* = 57) and C2-GBM (*n* = 109). The intersection of these two sets yielded 2555 tumor-related DEGs. Univariate Cox regression analysis of these DGEs identified 964 genes with prognostic significance. These DEGs were subjected to WGCNA, resulting in the identification of ten modules, each assigned a different color. Protein interaction network analysis highlighted genes involved in immune system and cell cycle processes. Hub genes identified for each cluster were C1-GBM (IGKV3-11, VAMP8, LAIR1, COL1A2, PLAUR) and C2-GBM (CKAP2L, PRSS51, MAST1, SOX6). (**B**) Module–module correlation heatmap from WGCNA. Each square shows the correlation between two modules (red, stronger; blue, weaker). Numbers in parentheses indicate the number of genes in each module: Magenta (*n* = 41), Yellow (*n* = 180), Turquoise (*n* = 159), Black (*n* = 46), Red (*n* = 7), Grey (*n* = 28), Brown (*n* = 39), Green (*n* = 45), Blue (*n* = 352), and Pink (*n* = 67). (**C**) Correlation of modules and hub genes with subtypes. The heatmap shows correlations between WGCNA modules and subtypes (C1 and C2) along with their corresponding hub genes. Correlation coefficients are color-coded: red for positive and green for negative correlations. Statistical significance is indicated as *** *p* < 0.001; ns indicates no significant difference. Modules and their hub genes are Magenta (IGKV3-11), Turquoise (LAIR1), Yellow (VAMP8), Black (COL1A2), Red (PLAUR), Brown (CKAP2L), Green (PRSS51), Blue (MAST1), and Pink (SOX6). (**D**,**E**) Protein–protein interaction analysis in the STRING database for the Yellow (**D**) and Brown (**E**) modules. (**F**) Violin plots of hub genes expression levels (Log_2_ FPKM) in TCGA-GBM samples. Genes include IGKV3-11, LAIR1, VAMP8, CKAP2L, COL1A2, MAST1, PLAUR, PRSS51, and SOX6. Statistical significance is denoted as **** *p* < 0.0001. (**G**) Heatmap of correlation between hub gene expression (RNA sequencing scores) and cancer stem cell (CSC) enrichment scores. Correlation coefficients are color-coded, with red for positive and blue for negative correlations. Statistical significance is denoted as * *p* < 0.05, ** *p* < 0.01, *** *p* < 0.001, **** *p* < 0.0001; ns indicates no significant difference. Hub genes include SOX6, PRSS51, PLAUR, MAST1, COL1A2, CKAP2L, VAMP8, IGKV3-11, and LAIR1. (**H**) Risk score analysis based on hub genes. Kaplan–Meier survival curve comparing patients stratified into low- and high-risk groups according to risk scores derived from hub gene expression (*p* < 0.001, HR = 2.33, log rank test). Blue and red lines indicate the low- and high- risk groups, respectively. Shaded areas denote the 95% confidence intervals.

We employed Weighted Correlation Network Analysis (WGCNA) to partition 964 prognostic, tumor-related genes into ten co-expression modules and to identify hub genes based high module membership (Figure 2A,B). After excluding the grey module (unassigned genes), module–trait correlation analysis showed that the Yellow and Turquoise modules were positively associated with C1-GBM, whereas the Brown module was associated with C2-GBM (Figure 2C). Protein–protein interaction analysis using the STRING database indicated genes in the Yellow module was enriched for immune system-related functions, while the Brown module was predominantly involved in cell cycle processes (Figure 2D,E).

Among the selected hub genes, immunoglobulin κ variable 3-11 (IGKV3-11), leukocyte-associated immunoglobulin-like receptor 1 (LAIR1), vesicle-associated membrane protein 8 (VAMP8), type I collagen α2 chain (COL1A2), and plasminogen activator urokinase receptor (PLAUR) were highly expressed in C1-GBM, whereas cytoskeleton-associated protein 2-like (CKAP2L), serine protease 51 (PRSS51), microtubule-associated serine/threonine kinase 1 (MAST1), and SRY-box transcription factor 6 (SOX6) showed elevated expression in C2-GBM (Figure 2F). In addition, the cancer stemness score (RNAss) was negatively correlated with LAIR1 and VAMP8 and positively correlated with CKAP2L and SOX6 (Figure 2G), indicating that these hub genes are linked to tumor stemness-related characteristics. Survival analysis further showed that higher expression of IGKV3-11, LAIR1, VAMP8, COL1A2, and PLAUR was associated with poorer overall survival, whereas higher SOX6 expression was significantly associated with improved prognosis (Supplementary Figure S2A), supporting their potential prognostic value. Finally, a risk score derived from the nine-gene signature stratified patients into high- and low-risk groups; the high-risk group exhibited a 2.33-fold higher hazard compared with the low-risk group (Figure 2H), highlighting the significance of these signatures for risk prediction.

### 3.3. Classification Model Utilizing Machine Learning on Subtype-Specific Hub Genes

To apply the novel subtypes to the external dataset, we constructed a neural network-based classifier. Neural networks can capture nonlinear relationships and learn high-level representations from omics data [39,40], and they have been widely used for cancer classification tasks [41].

**Figure 3:**
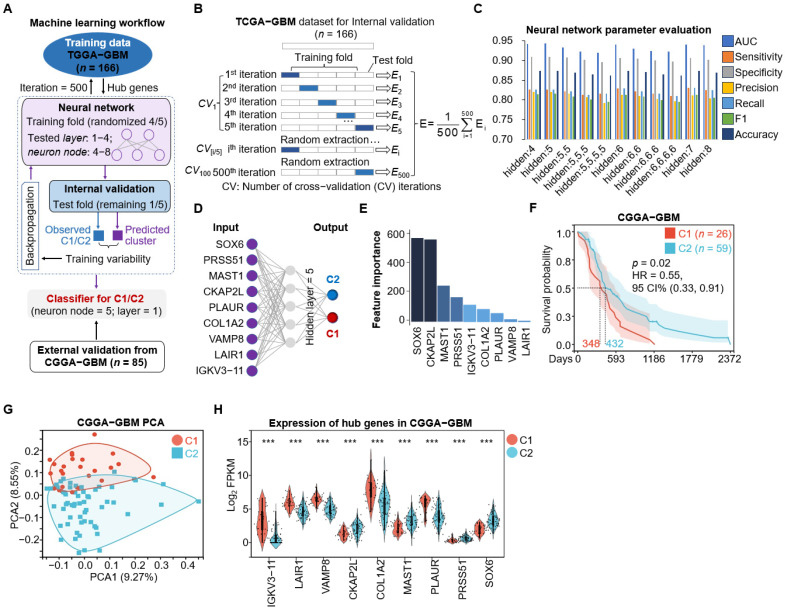
Classification model utilizing machine learning on subtype-specific hub genes. (**A**) Machine learning workflow for subtype classification using neural networks. This diagram outlines the process of training and validating a neural network for classifying subtypes C1 and C2 subtypes using the TCGA-GBM dataset, with external validation using the CGGA-GBM dataset. Key steps include the following: (1) Training data from the TCGA-GBM dataset includes 166 samples, utilizing hub genes as input features over 500 iterations. (1) (2) Neural network configuration involves using four out of five folds for training in each iteration, testing various configurations of hidden layers (1 to 4) and neuron nodes (4 to 8) to determine the optimal network architecture. (3) Internal validation with the remaining fifth folds is used to compare observed subtypes with predicted clusters, employing backpropagation to adjust the network weights based on the validation outcomes. (4) The final classifier for C1/C2 classification consists of one hidden layer with five neurons. (5) External validation using the CGGA-GBM dataset includes 85 samples. (**B**) Internal validation of the TCGA-GBM dataset (*n* = 166) using 5-fold cross-validation. The dataset (*n* = 166) undergoes five-fold cross-validation, where one fold serves as the test set in each iteration and the others as training sets, generating validation errors (E1 to E5). This process is repeated over 500 iterations, with the mean error (E) calculated from all iterations. (**C**) Bar plot of neural networks performance metrics with different hidden layer and neuron counts. (**D**) Neural network architecture for subtype classification. The network consists of an input layer with 9 features (SOX6, PRSS1, MAST1, CKAP2L, PLAUR, COL1A2, VAMP8, LAIR1, and IGKV3-11), a single hidden layer with 5 neurons, and an output layer classifying into C1 (red) and C2 (blue). (**E**) Bar plot of hub genes features importance in the TCGA-GBM dataset. The importance of genes is determined by the BP neural network classifier. SOX6 ranks as the most influential, followed by CKAP2L, MAST1, PRSS51, IGKV3-11, COL1A2, PLAUR, VAMP8, and LAIR1. The color intensity of the bars reflects gene feature importance (higher importance is shown with darker colors). (**F**) Kaplan–Meier survival curves for classified clusters. The survival curves for cluster C1 (red, *n* = 26) and cluster C2 (blue, *n* = 59) are obtained using the BP neural network classifier on the external CGGA-GBM dataset (*p* = 0.02, HR = 0.55, log rank test), with shaded areas indicating the 95% confidence intervals. The dashed lines indicate the median survival time. (**G**) PCA of C1 and C2 subtypes in the external CGGA-GBM dataset classified by the BP neural network classifier, illustrating the distribution of GBM samples. The *x*-axis (PCA1) and *y*-axis (PCA2) represent the first and second principal components, respectively. (**H**) Violin plots of hub genes expression levels (Log_2_ FPKM) in CGGA-GBM samples. The genes include IGKV3-11, LAIR1, VAMP8, CKAP2L, COL1A2, MAST1, PLAUR, PRSS51, and SOX6, which demonstrate consistent expression patterns in TCGA-GBM. Statistical significance is indicated as *** *p* < 0.001.

We developed a backpropagation (BP) neural network model to classify the C1 and C2 subtypes (Figure 3A). The model was trained on the TCGA-GBM cohort (*n* = 166) using repeated 5-fold cross-validation over 500 iterations. In each iteration, samples were randomly partitioned into five folds; four folds (approximately 80%) were used for training and the remaining fold (approximately 20%) was held out for internal validation. The nine hub genes were used as input features, and predicted labels were compared with the consensus clustering-defined C1/C2 assignments to evaluate model performance (Figure 3B). We evaluated multiple network architectures by varying the number of hidden layers (one to four) and the number of neurons per layer (four to eight), and optimized model weights using the backpropagation algorithm to minimize classification error. The final classifier consisted of a single hidden layer with five neurons and achieved a ROC AUC of 0.94, indicating strong discriminative performance (Figure 3C,D). Feature contribution analysis of the nine-gene signature showed that SOX6 had the highest contribution (Figure 3E). The trained classifier was then applied to the independent CGGA-GBM cohort (*n* = 85), stratifying patients into C1 (*n* = 26) and C2 (*n* = 59). Survival analysis and PCA in the CGGA cohort, together with subtype-specific hub gene expression patterns and their associations with prognosis, were consistent with the trends observed in the TCGA training cohort (Figure 3F−H and Supplementary Figure S3A), supporting the robustness of the classifier for external subtype prediction.

### 3.4. Differential Expression Genes Analyses Indicate Characteristics of C1 and C2 Subtypes

**Figure 4:**
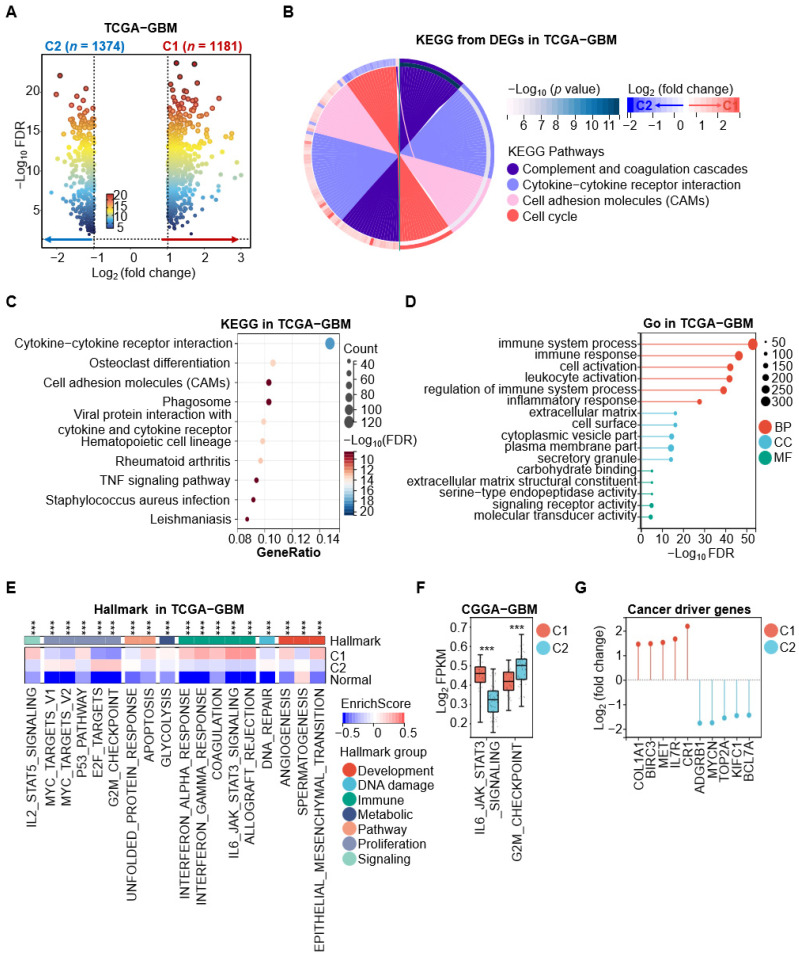
Differential expression genes analyses indicate characteristics of C1 and C2 subtypes. (**A**) Volcano plot of differential expression genes (DEGs) between C1 (*n* = 1181) and C2 (*n* = 1374) subtypes in the TCGA-GBM dataset. The *x*-axis represents the Log_2_ FC in gene expression between the two subtypes, while the *y*-axis shows the negative logarithm (base 10) of the false discovery rate (−Log_10_ FDR). Each dot represents a gene, color-coded by expression level from blue (low expression) to red (high expression). Vertical dashed lines indicate the threshold for significant differential expression (|Log_2_ FC| > 1). Genes to the right of the dashed line are upregulated in C1, while genes to the left are upregulated in C2. (**B**) Circular plot of the KEGG pathway analysis of DEGs between C1 and C2 subtypes in the TCGA-GBM dataset. (**C**) Dot plot of KEGG pathway enrichment analysis of DEGs. The x-axis represents the gene ratio, which measures the proportion of DEGs involved in a specific pathway relative to the total number of DEGs. The y-axis lists the significantly enriched KEGG pathways. Each dot represents a KEGG pathway, with the dot size indicating the count of DEGs involved. (**D**) Gene Ontology (GO) enrichment analysis of DEGs in the TCGA-GBM dataset. The x-axis represents the −Log_10_ FDR value, indicating enrichment significance. The y-axis lists the significantly enriched GO terms categorized into Biological Processes (BPs, red), Cellular Components (CCs, blue), and Molecular Functions (MFs, green). Dot size reflects the number of DEGs associated with each GO term, with larger dots indicating a higher count of genes. (**E**) Hallmark gene set analysis comparing C1 and C2 subtypes with normal controls. The *x*-axis represents different hallmark pathways and the *y*-axis lists the samples categorized as C1, C2, and normal. Statistical significance denoted as *** *p* < 0.001. (**F**) Evaluation of pathway enrichment scores. This panel examine the enrichment score for the IL6_JAK_STAT3_SIGNALING and G2/M_CHECKPOINT pathways in the CGGA-GBM dataset. Statistical significance denoted as *** *p* < 0.001. (**G**) Comparative analysis of cancer driver gene abundance between C1 and C2 subtypes in the TCGA-GBM dataset, with the bar plot showing the Log_2_ FC in the expression of cancer driver genes between C1 (red) and C2 (blue) subtypes.

Given the distinct prognostic differences between the C1 and C2 subtypes, we performed differential expression analysis to identify genes with significant differential expression between C1-GBM and C2-GBM (C1 vs. C2). The analysis identified 6598 differentially expressed genes (DEGs) with a false discovery rate (FDR) < 0.05 and a fold change > |1.5| (Figure 2A). We then retained only those DEGs that also showed significant tumor–normal differences, resulting in 2555 tumor-related, subtype-specific DEGs that met both criteria (tumor vs. normal: FDR < 0.05, fold change > |1.5|; C1 vs. C2: FDR < 0.05, fold change > |1.5|). Among these, 1181 tumor-related genes were upregulated in C1-GBM and 1374 were upregulated in C2-GBM (Figure 4A, Table S1).

Pathway and Gene Ontology (GO) enrichment analysis was performed using the clusterprofiler R package with FDR < 0.05 as the significance cutoff. DEGs upregulated in C1-GBM were primarily enriched in the cytokine-cytokine receptor interaction, whereas DEGs upregulated in C2-GBM were mainly enriched in the cell cycle pathway (Figure 4B). Enrichment analysis of all 2555 DEGs further indicated broad involvement in immune-related pathways and biological processes (Figure 4C,D). In addition, analysis of MSigDB hallmark gene sets associated with cancer development, immunity, and signaling pathways, revealed significant differences in enrichment scores among C1-GBM, C2-GBM, and normal samples [22]. Specifically, C1-GBM showed higher enrichment of immune-related hallmarks, whereas C2-GBM exhibited stronger enrichment of proliferation-related hallmarks (Figure 4E). These patterns were further supported in the CGGA cohort, particularly for the IL6_JAK_STAT3_SIGNALING and G2/M_CHECKPOINT pathways, consistent with the trends observed in TCGA (Figure 4F).

Additionally, we investigated specific genes driving oncogenesis of C1-GBM and C2-GBM. In C1-GBM, key tumor driver genes included BIRC3 (Baculoviral IAP Repeat Containing 3), MET (MET Proto-Oncogene, Receptor Tyrosine Kinase), COL1A1 (Collagen Type I Alpha 1 Chain), CR1 (Complement C3b/C4b Receptor 1), and IL7R (Interleukin 7 Receptor). Conversely, in C2-GBM, distinct oncogenic roles were played by ADGRB1 (Adhesion G Protein-Coupled Receptor B1), MYCN (MYCN Proto-Oncogene, BHLH Transcription Factor), TOP2A (DNA Topoisomerase II Alpha), KIFC1 (Kinesin Family Member C1), and BCL7A (BAF Chromatin Remodeling Complex Subunit BCL7A) (Figure 4G). Overall, this study revealed distinct tumor characteristics between the C1-GBM and C2-GBM, with C1-GBM primarily linked to immune-related processes and C2-GBM to cell proliferation-related processes.

### 3.5. C1-GBM Exhibits an Immune-Infiltrated (“Hot”) Tumor Microenvironment with Checkpoint Upregulation

**Figure 5:**
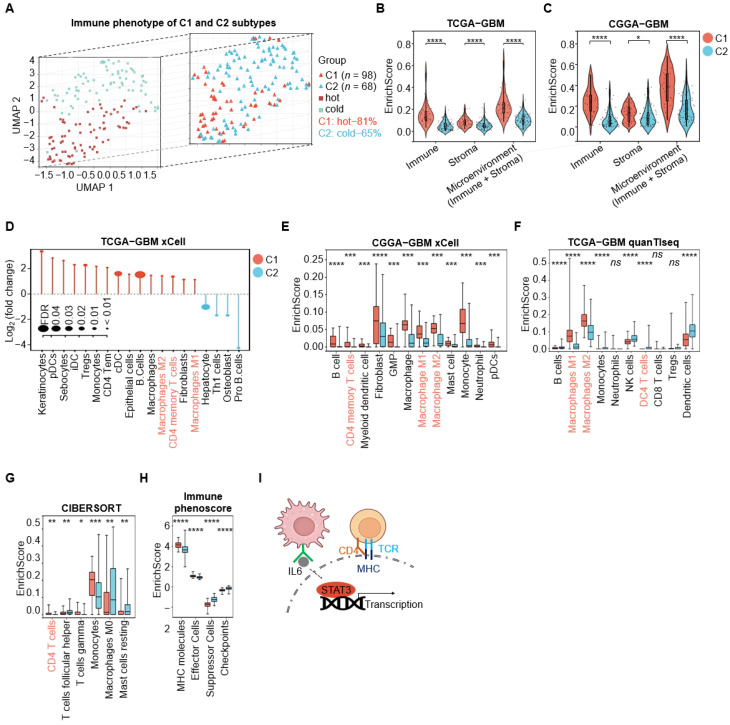
C1-GBM exhibits an immune-infiltrated (“hot”) tumor microenvironment with checkpoint upregulation. (**A**) Immune phenotype of C1 and C2 subtypes in the TCGA-GBM dataset. The left panel uses UMAP (Uniform Manifold Approximation and Projection) for dimensionality reduction to display the distribution of “hot” and “cold” tumors, with “hot” tumors in red squares and “cold” tumors in green squares. The right panel displays C1 and C2 subtypes, using the same UMAP dimensions: C1 is in red triangles and C2 is in blue triangles. (**B**,**C**) Violin plots of the enrichment scores for immune, stromal, and microenvironment (immune + stroma) components in C1 (red) and C2 (blue) subtypes in both the TCGA-GBM and CGGA-GBM datasets. Statistical significance denoted as * *p* < 0.05, **** *p* < 0.0001. (**D**) Differential cell type enrichment in the TCGA-GBM dataset via xCell. The *y*-axis represents the log_2_ FC in cell type abundance, with positive values indicating higher enrichment in C1 and negative values indicating higher enrichment in C2. The *x*-axis lists the cell types analyzed, and dot size represents FDR, with smaller dots indicating lower FDR values. (**E**) Differential cell type enrichment in the CGGA-GBM dataset via xCell. The *y*-axis represents the enrichment score, while the *x*-axis lists the cell types analyzed. Statistical significance denoted as *** *p* < 0.001, **** *p* < 0.0001. (**F**) Differential cell type enrichment in the TCGA-GBM dataset via quanTIseq. The *y*-axis represents the enrichment score, while the *x*-axis lists the cell types analyzed. Statistical significance denoted as **** *p* < 0.0001; ns indicates no significant difference. (**G**) Differential cell type enrichment in the TCGA-GBM dataset via CIBERSORT. Statistical significance denoted as * *p* < 0.05, ** *p* < 0.01, *** *p* < 0.001. (**H**) Immune phenoscore enrichment in C1 and C2 subtypes in the TCGA-GBM dataset. The y-axis represents the enrichment score for immune components, including MHC molecules, effector cells, suppressor cells, and checkpoints. Statistical significance denoted as **** *p* < 0.0001. In subfigures (**E**–**H**), red and blue indicate C1 and C2, respectively. In subfigures (**D**–**G**), cell types shown in red text denote overlapping findings identified across different methods. (**I**) Potential mechanisms of the C1 subtype.

This study reveals that C1-GBM is preferentially enriched for immune-related pathways, whereas C2-GBM is more associated with cell cycle pathways through differential expression and enrichment analyses. To further characterize the biological basis of these differences, we investigated the tumor microenvironment (TME), a complex system consisting of various cells, molecules, and structures around a tumor which can shape tumor growth, invasion, and immune escape [42]. Chen et al. classified tumors into three immune phenotypes based on the degree of immune cell infiltration: immune-inflamed, immune-excluded, and immune-desert phenotypes [43]. Immune-inflamed tumors are often referred to as “hot” tumors, whereas immune-excluded and immune-desert tumors are commonly considered “cold” tumors [42,44]. In this study, we operationally defined “hot” tumors as those showing overall high immune cell infiltration and “cold” tumors as those with low infiltration. Among tumors with evaluable immune cell composition estimates (*n* = 63), 51 (81%) in C1-GBM exhibited substantial immune cell enrichment (Figure 5A), supporting an immune-inflamed (“hot”) phenotype.

We used four complementary computational methods (xCell, CIBERSORT, quanTISeq, and Immunophenoscore) to quantify immune infiltration and identify immune cell types. Across both TCGA-GBM and CGGA-GBM cohorts, C1-GBM consistently exhibited significantly higher immune scores than C2-GBM (Figure 5B,C). In particular, CD4^+^ T cells (supported by xCell, CIBERSORT, and quanTIseq) and macrophage subsets (M1 and M2 macrophages estimated by xCell and quanTISeq) were significantly more abundant in C1-GBM (Figure 5D,F,G). These findings were validated in the CGGA-GBM cohort, which also showed enrichment of CD4^+^ memory T cells, M1 macrophages, and M2 macrophages in C1-GBM (Figure 5E). Consistently, hallmark pathway analysis indicated that the IL6_JAK_STAT3_SIGNALING pathway was most enriched in C1-GBM, accompanied by elevated expression of key genes such as STAT3 (Signal Transducer and Activator of Transcription 3) and JAK1 (Janus Kinase 1) (Supplementary Figure S4A). IL-6 (Interleukin-6), a macrophage-secreted factor, can activate STAT3 to promote gene transcription [45,46].

Furthermore, immune phenotype analysis revealed that C1-GBM had higher enrichment of major histocompatibility complex (MHC) molecules and effector cell signatures, together with increased checkpoint-related signals, while showing lower suppressor-cell signals (Figure 5H). MHC molecules are essential for antigen presentation and the initiation of adaptive immune responses [47,48]. Correspondingly, MHC pathway-associated genes, including HLA-C (Major Histocompatibility Complex, Class I, C), HLA-DPA1 (Major Histocompatibility Complex, Class II, DP Alpha 1), HLA-DPB1 (Major Histocompatibility Complex, Class II, DP Beta 1), HLA-DQA1 (Major Histocompatibility Complex, Class II, DQ Alpha 1), HLA-DQA2 (Major Histocompatibility Complex, Class II, DQ Alpha 2), HLA-DRB5 (Major Histocompatibility Complex, Class II, DR Beta 5), and HLA-DRB6 (Major Histocompatibility Complex, Class II, DR Beta 6), were significantly upregulated in C1-GBM (Supplementary Figure S4B). Additionally, genes related to PD1 pathway, such as BATF (Basic Leucine Zipper ATF-Like Transcription Factor), HLA-DRB1 (Major Histocompatibility Complex, Class II, DR Beta 1), CD3D (CD3 Delta Subunit Of T-Cell Receptor Complex), LCK (LCK Proto-Oncogene, Src Family Tyrosine Kinase), PDCD1LG2 (Programmed Cell Death 1 Ligand 2), CD3E (CD3 Epsilon Subunit Of T-Cell Receptor Complex), CD3G (CD3 Gamma Subunit Of T-Cell Receptor Complex), CD8B (CD8 Subunit Beta), NFATC2 (Nuclear Factor Of Activated T Cells 2), and PDCD1 (Programmed Cell Death 1), were also significantly overexpressed in C1-GBM, suggesting their potential as therapeutic targets (Supplementary Figure S4C). In summary, C1-GBM is characterized by a highly immune-infiltrated microenvironment, with MHC-mediated antigen presentation, IL-6 secretion by macrophages, and the PD1 pathway playing critical roles in coordinating immune responses (Figure 5I), collectively emphasizing its distinct immunological landscape.

### 3.6. C2-GBM Exhibits a Tumor-Intrinsic Proliferative State with G2/M Checkpoint Activation

**Figure 6:**
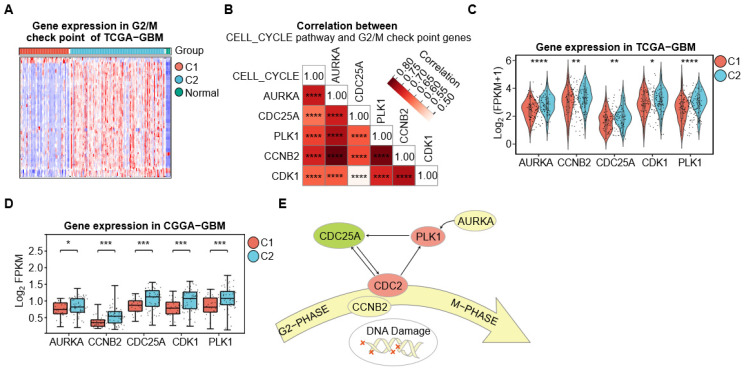
C2-GBM exhibits a tumor-intrinsic proliferative state with G2/M checkpoint activation. (**A**) Gene expression heatmap of DEGs involved in G2/M checkpoint. Red, blue, and green represent C1, C2, and Normal, respectively. (**B**) Correlation study between CELL_CYCLE genes and G2/M checkpoint genes. Statistical significance denoted as **** *p* < 0.0001. (**C**,**D**) Comparative expression analysis of five key genes at the G2/M checkpoint in TCGA-GBM and CGGA-GBM. Statistical significance is denoted as * *p* < 0.05, ** *p* < 0.01, *** *p* < 0.001, **** *p* < 0.0001. (**E**) Potential mechanisms associated with the C2 subtype.

Analysis of hallmark gene sets revealed that the C2-GBM had the highest enrichment score for the G2/M checkpoint relative to C1-GBM (Figure 4E). The G2/M checkpoint pathway is essential for cell cycle progression and plays a significant role in cancer development, especially in high-risk populations [49]. We retrieved the hallmark G2/M checkpoint gene set (200 genes) and found that 82 genes were significantly upregulated in C2-GBM compared with both C1-GBM and normal samples, indicating pronounced activation of the G2/M checkpoint pathway in C2-GBM (Figure 6A, Table S1). Previous studies have reported that during the M phase, the kinase Aurora A (AURKA) and its cofactor Bora jointly activate PLK1, which subsequently triggers CDC25 phosphatase and the downstream activation of CDC2 (CDK1), forming a feedback loop that efficiently propels cells into mitosis [49]. Our analysis revealed a high positive correlation between the expression of AURKA, CDC25A, PLK1, CCNB2, and CDK1, all of which were highly expressed in C2-GBM (Figure 6C). Moreover, the expression of these genes was positively correlated with KEGG cell cycle pathway enrichment score (Figure 6B), supporting their association with enhanced G2/M checkpoint activity and proliferative capacity. This pattern was consistent in the CGGA-GBM cohort for the C2-GBM, further reinforcing the connection between these genes and G2/M checkpoint activation (Figure 6D). Collectively, these findings indicate that C2-GBM contributes to tumor growth through enhanced activation of the G2/M checkpoint pathway, underscoring its critical role in cell cycle regulation and tumor progression (Figure 6E).

### 3.7. Establishment of a GBM Survival Model

**Figure 7:**
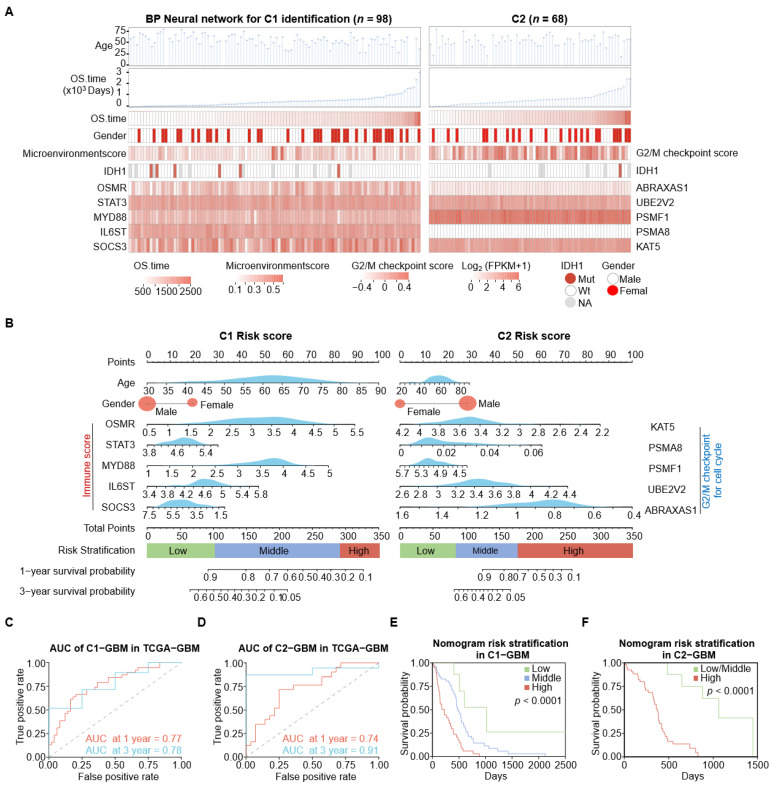
Establishment of a GBM survival model. (**A**) Comparison of clinical and molecular characteristics between C1 (left, *n* = 98) and C2 (right, *n* = 68) subtypes, sorted by overall survival (OS) time. The top panel displays age distribution, followed by OS time in days, with a heatmap indicating OS duration (darker shades indicate longer survival). Subsequent panels show gender distribution and IDH1 mutation status. The heatmap displays microenvironment scores, gene expression levels for IL6 pathway genes (OSMR, STAT3, MYD88, IL6ST, and SOCS3) in C1, and G2/M checkpoint pathway genes (ABRAXAS1, UBE2V2, PSMF1, PSMA8, and KAT5) in C2. (**B**) Constructed nomogram model predicting the 1- and 3-year survival probabilities for patients with C1 (left) and C2 (right) risk scores. Density plots display distribution of age, IL6 pathway genes (OSMR, STAT3, MYD88, IL6ST, and SOCS3) in C1, and G2/M checkpoint pathway genes (ABRAXAS1, UBE2V2, PSMF1, PSMA8, and KAT5) in C2. Distribution of category variable (gender) is reflected by the size of the circle. Risk stratification was performed using the R package maxstat to determine optimal cut-off points for total points derived from a Cox model, dividing patients into low-, middle-, and high-risk groups. These cut-offs were calculated through stepwise maximally selected rank statistics, ensuring alignment with survival outcomes. (**C**,**D**) Analysis of the area under the curve (AUC) for 1-year and 3-year prognostic predictions using the nomogram model of C1/C2 in the TCGA-GBM dataset. The dashed diagonal line indicates the no-discrimination reference (random classifier; AUC = 0.5). (**E**,**F**) TCGA-GBM patients in the cohort at different risks stratified according to the nomogram of C1/C2 subtype. In C1-GBM, the sample sizes were as follows: low-risk group (*n* = 8), middle-risk group (*n* = 47), and high-risk group (*n* = 39). In C2-GBM, the middle-risk group with one patient was merged into the low-risk group, resulting in sample sizes of low-risk group (*n* = 10) and high-risk group (*n* = 52). NA values were removed when calculating the cutoff values for low-, middle-, and high-risk groups in both C1-GBM and C2-GBM subtypes. This resulted in a reduction in the sample sizes allocated to each risk group.

C1-GBM is characterized by an immune-infiltrated microenvironment with significant IL6 pathway activity, whereas C2-GBM is marked by tumor driver and activation of G2/M checkpoint for the cell cycle. To develop subtype-specific survival prediction models based on C1/C2 assignments as predicted by the BP neural network classifier, we explored clinical and molecular characteristics between C1-GBM (*n* = 98) and C2-GBM (*n* = 68) subtypes (Figure 7A). We employed correlation analysis between IL6 pathway-related genes and overall survival (OS) time in C1-GBM, as well as G2/M checkpoint pathway-related genes and OS time in C2-GBM, to select candidate variables (*p* < 0.05). According to the correlation analysis results, the model includes age, gender, log_2_ (FPKM + 1) gene expression level of OSMR, STAT3, MYD88, IL6ST, and SOCS3 for C1-GBM and ABRAXAS1, UBE2V2, PSMF1, PSMA8, and KAT5 for C2-GBM. We then constructed subtype-specific nomograms using variables screened (Figure 7B). Model performance was evaluated using time-dependent ROC analysis. In the TCGA-GBM training cohort, the time-dependent AUC values were above 0.7 for predicting 1-year and 3-year OS in both the C1-GBM and C2-GBM models (Figure 7C,D), demonstrating the nomogram’s effective discrimination capabilities. We further implemented risk stratification based on the total nomogram points. For C1-GBM, patients were classified into three risk groups: low-risk (total points < 102), middle-risk (102 ≤ total points < 291), and high-risk (total points ≥ 291). Similarly, patients in C2-GBM were stratified into three risk groups: low-risk (total points < 83), middle-risk (83 ≤ total points < 176), and high-risk (total points ≥ 176). The Kaplan–Meier analyses showed significant discrimination among the risk groups in C1-GBM and C2-GBM (*p* < 0.0001, log-rank test; Figure 7E,F).

### 3.8. Sensitive Drug Selection for Immune-Related C1 and Proliferative C2 Subtypes

**Figure 8:**
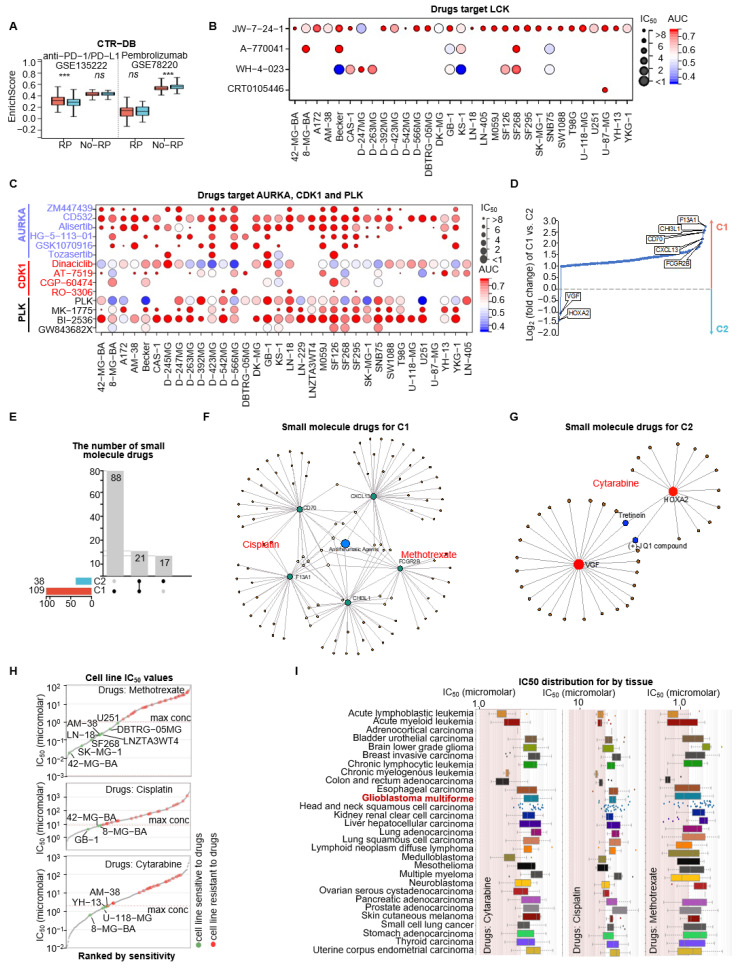
Sensitive drug selection for immune-related C1 and tumor driver-related C2 subtypes. (**A**) Drug sensitivity (responsive) and resistance gene sets (non-responsive) for Pembrolizumab and anti-PD-1/PD-L1 from CTR-DB. The box plot depicts the enrichment scores for response prediction to anti-PD-1/PD-L1 therapy based on two gene expression datasets: GSE135222 and GSE78220. The groups are divided into responders (RPs) and non-responders (No-RPs) to the treatment. Statistical significance is denoted as *** *p* < 0.001 and ns indicates no significant difference. Red and blue boxes represent C1 and C2, respectively. The dash line separates the two datasets. (**B**) Evaluation of IC_50_ values and area under the curve (AUC) for drugs targeting LCK in GBM cell lines from the GDSC database. (**C**) Assessment of IC_50_ (filtered by maximum concentration) and AUC for drugs targeting AURKA, CDK1, and PLK in GBM cell lines using the GDSC database. (**D**) Ranking of Log_2_ FC of prognostic DEGs between C1 and C2 subtypes. The y-axis represents the Log_2_ FC of C1 vs. C2, with positive values indicating higher expression in C1 and negative values indicating higher expression in C2. Notably expressed genes include F13A1, CHI3L1, CD70, CXCL13, and FCGR2B, upregulated in C1, and VGF and HOXA2, upregulated in C2. (**E**) The number of small-molecule drug interactions for C1 (red) and C2 (blue). The grey bars indicate the total number of drugs: 88 drugs unique to C1, 21 drugs common to both subtypes, and 17 drugs unique to C2. The left bar plot provides the total number of drugs associated with each subtype: 109 for C1 and 38 for C2. (**F**,**G**) Gene targets of small-molecule drugs in C1 and C2. (**H**) Presentation of IC_50_ values for GBM cell lines treated with Methotrexate, Cisplatin, and Cytarabine. (**I**) Distribution analysis of IC_50_ values by tissue type in GBM cell lines for Methotrexate, Cisplatin, and Cytarabine (IC_50_: 50% inhibitory concentration; max conc: maximum screening concentration in µM; AUC: area under the curve).

Given the distinctive features of the two subtypes, we aimed to identify candidate drugs with potential subtype-specific efficacy. First, we retrieved pembrolizumab- and anti PD-1/PD-L1 response-related gene sets from CTR-DB and grouped them into signatures associated with drug sensitivity (response) and drug resistance (non-response). Enrichment analysis showed that C1-GBM displayed higher enrichment of the response signature, suggesting a greater likelihood of sensitivity to anti PD-1/PD-L1 therapy, whereas C2-GBM showed higher enrichment of the non-response signature, indicating potential resistance to pembrolizumab (Figure 8A).

Next, to target subtype-specific pathways, we explored the Genomics of Drug Sensitivity in Cancer (GDSC) resource for compounds directed against key DEGs in the PD1 pathway (predominant in C1) and five DEGs in the G2/M checkpoint pathway (predominant in C2) [50]. Our analysis highlighted JW-7-24-1, which targets LCK, as effective in 35 GBM cell lines, suggesting its potential against the PD1 pathway in C1-GBM (Figure 8B, Table S2). For C2-GBM, CD532 (targeting AURKA) and BI-2536 (targeting PLK1) showed efficacy in over 30 GBM cell lines, indicating their suitability for this subtype (Figure 8C, Table S2).

Finally, univariable Cox regression identified 138 DEGs associated with worse prognosis (HR > 1, *p* < 0.05) (Figure 8D, Table S1). We further prioritized subtype-specific high-expression prognostic DEGs as potential targets for small-molecule discovery, including F13A1 (Coagulation Factor XIII A Chain), CHI3L (Chitinase 3 Like 1), CXCL3 (C-X-C Motif Chemokine Ligand 3), FCGR2B (Fc Gamma Receptor IIb) in C1-GBM (log_2_ FC > 2), and VGF (VGF Nerve Growth Factor Inducible) and HOXA2 (Homeobox A2) in C2-GBM (log_2_ FC < −1). This led to the identification of 109 candidate compounds for C1 and 38 for C2 after excluding 21 overlapping compounds (Figure 8E, Table S3). Drug sensitivity analysis based on GDSC data prioritized Methotrexate and Cisplatin for C1-associated targets, whereas Cytarabine was prioritized for C2-GBM (Figure 8F–H, Table 2). Notably, these drugs have also demonstrated efficacy against acute lymphoblastic leukemia and cervical squamous cell carcinoma (Figure 8I).

## 4. Discussion

In this study, we created pathway-level activity profiles derived from MSigDB gene sets and applied unsupervised consensus clustering to classify GBM into two prognostically distinct subtypes. We further constructed a BP neural network classifier using a nine-gene signature (including IGKV3-11, LAIR1and SOX6) to enable subtype prediction in an independent cohort. Collectively, our results support a pathway-informed framework for GBM stratification that links clinical outcome to both tumor-intrinsic programs and the tumor immune microenvironment.

A key methodological feature of our work is the pathway-based representation of transcriptomic data. Compared with single-gene classifiers, pathway-level signatures can be more robust and biologically interpretable because they capture coordinated programs that underpin tumor behavior [17,19,20,51]. Using MSigDB signatures, our subtype solution showed clear survival separation (*p* < 0.0001, log-rank test) and demonstrated concordance with established transcriptomic categories, with C1-GBM more aligned with mesenchymal-like patterns and C2-GBM more aligned with classical- and proneural-like patterns [6,7]. This concordance suggests that the proposed subtypes capture biologically meaningful variation while providing improved prognostic resolution.

Mechanistically, the two subtypes appear to be driven by different dominant programs. C1-GBM is characterized by an immune-infiltrated microenvironment and prominent cytokine-associated signaling, with IL6–JAK–STAT3 signaling emerging as a representative pathway [45,46]. In line with an immune-inflamed (“hot”) phenotype, C1-GBM shows enrichment of immune cell signatures, including CD4+ T cells and macrophage-related programs. Memory CD4^+^ T cells can shape antitumor immunity by coordinating immune responses, and antigen presentation through major histocompatibility complex (MHC) molecules is critical for T cell activation and immune coordination [47,48,52,53]. The IL6-JAK-STAT3 axis provides mechanistic link between inflammatory signaling and tumor progression, as IL-6 can activates Janus kinase (JAK) enzymes to promote STAT3 phosphorylation and nuclear translocation, thereby regulating transcriptional programs involved in inflammation, survival, and immune modulation [45,46,54,55]. Notably, the immune-infiltrated state in C1-GBM is accompanied by checkpoint-associated signals, which is consistent with adaptive immune resistance and may reflect immune exhaustion features. This biological context supports the potential relevance of PD-1/PD-L1-directed strategies for a subset of C1-GBM patients while also emphasizing that immune infiltration alone does not necessarily translate into effective tumor control.

In contrast, C2-GBM exhibits a tumor-intrinsic proliferative program with low immune infiltration and strong activation of cell cycle regulation, including the G2/M checkpoint. Elevated activity of mitotic entry pathways suggests dependence on core cell cycle machinery, in which the Cyclin B–CDK1 complex plays a central role in driving the G2-to-M transition [49,56]. In addition, C2-GBM shows features consistent with oncogenic driver activity. Tumor drivers are genetic alterations that confer growth advantages and shape tumor evolution under selective pressures. In this context, increased MYCN expression, a MYC-family transcription factor implicated in proliferation and differentiation programs in multiple cancers including neuroblastoma, may contribute to the aggressive proliferative phenotype of C2-GBM [57,58]. Together, these observations support the interpretation that C2-GBM progression is primarily governed by proliferative and cell cycle programs, suggesting that therapeutic hypotheses for this subtype may prioritize targeting mitotic checkpoint vulnerabilities and oncogenic growth signaling rather than immune modulation.

In summary, we propose a pathway-informed classification of GBM into two prognostically distinct subtypes. C1-GBM represents an immune-infiltrated subtype with prominent inflammatory signaling and checkpoint-associated features, whereas C2-GBM represents a proliferation-associated subtype with strong G2/M checkpoint activity and tumor-intrinsic growth programs. This framework may facilitate improved prognostic stratification and support the development of subtype-tailored therapeutic strategies.

## 5. Conclusions

In this study, we employed pathway-based unsupervised clustering to classify GBM patients into two prognostically distinct subtypes: C1-GBM (immunosuppression-driven), which exhibits mesenchymal-like features and an immune-infiltrated microenvironment with checkpoint-associated signals, and C2-GBM (tumor inherent-driven), which shows proneural-like features and a proliferation-associated program with prominent cell cycle activity. C1-GBM displayed elevated immune microenvironment scores and immunological activity, consistent with an immune-inflamed (“hot”) phenotype, and showed enrichment patterns suggestive of potential responsiveness to anti-PD-1/PD-L1 immunotherapy. In contrast, C2-GBM exhibited lower immune infiltration and stronger activation of cell-cycle programs, including the G2/M checkpoint. To facilitate rapid and accurate diagnosis and classification, we constructed a BP neural network classifier based on a nine-gene signature, including IGKV3-11, LAIR1, VAMP8, COL1A2, PLAUR, CKAP2 L, MAST1, PRSS51, and SOX6. Moreover, we constructed subtype-specific prognostic models to estimate survival risk for patients classified as C1-GBM or C2-GBM. Based on the distinctive characteristics of these subtypes, we recommend small-molecule chemotherapy, specifically Methotrexate and Cisplatin for C1-GBM, and Cytarabine for C2-GBM. Overall, our study presents a pathway-informed framework for GBM classification and risk prediction, and it may facilitate future efforts toward subtype-tailored precision therapy.

## Figures and Tables

**Table 1:** Clinical features of C1 and C2 subtypes.

Characteristics	C1 (*n* = 57)	C2 (*n* = 109)	Total (*n* = 166)	*p*-Value
OS time				*p* < 0.001
Mean ± SD	352.88 ± 333.62	466.52 ± 406.59	427.50 ± 385.89	
Median	313.00	425.00	360.00	
Age				
Mean ± SD	62.19 ± 12.15	58.71 ± 13.41	59.94 ± 13.04	ns
Median	62.77	60.01	60.80	
Gender				ns
Male	37 (65%)	70 (64%)	107	
Female	20 (35%)	39 (36%)	59	
GeneExp Subtype				*p* < 0.001
Classical	4 (7%)	37 (34%)	41	
Mesenchymal	35 (61%)	21 (19%)	56	
Neural	15 (26%)	13 (12%)	28	
Proneural	1 (2%)	37 (34%)	38	
DNA methylation				*p* < 0.001
Cluster 1	11 (19%)	3 (3%)	14	
Cluster 2	13 (23%)	16 (15%)	29	
Cluster 3	12 (21%)	20 (18%)	32	
Cluster 4	1 (2%)	24 (22%)	25	
Cluster 5	0	8 (7%)	8	
Cluster 6	0	13 (12%)	13	

ns: no significant difference.

**Table 2:** The cell line IC_50_ values of GBM cell lines for Methotrexate, Cisplatin, and Cytarabine.

Cell Line	TCGA Tumor	IC_50_ ^a^ (Filtered by Max Conc ^b^)	AUC ^c^	Drugs
42-MG-BA	GBM	0.02	0.44	Methotrexate
SK-MG-1	GBM	0.05	0.54	Methotrexate
SF268	GBM	0.10	0.63	Methotrexate
LN-18	GBM	0.19	0.72	Methotrexate
AM-38	GBM	0.19	0.71	Methotrexate
LNZTA3WT4	GBM	0.22	0.73	Methotrexate
DBTRG-05MG	GBM	0.39	0.72	Methotrexate
U251	GBM	0.84	0.84	Methotrexate
GB-1	GBM	3.95	0.81	Cisplatin
42-MG-BA	GBM	7.01	0.89	Cisplatin
8-MG-BA	GBM	7.59	0.91	Cisplatin
8-MG-BA	GBM	0.63	0.79	Cytarabine
U-118-MG	GBM	1.51	0.81	Cytarabine
YH-13	GBM	1.76	0.85	Cytarabine
AM-38	GBM	1.94	0.86	Cytarabine

^a^: 50% inhibitory concentration. ^b^: Max screening concentration (µM). ^c^: the area under the curve.

## Data Availability

The original contributions presented in this study are included in the article/Supplementary Material. Further inquiries can be directed to the corresponding authors.

## Supplementary Materials

The following supporting information can be downloaded at https://www.mdpi.com/article/10.3390/cimb48010103/s1.
