# Supplementary material for: Identification of Tumor- and Immunosuppression-Driven Glioblastoma Subtypes Characterized by Clinical Prognosis and Therapeutic Targets
Source: Curr Issues Mol Biol. 2026 Jan 19;48(1):103. doi: 10.3390/cimb48010103 (PMC12839865; doi:10.3390/cimb48010103)
Supplement: Supplementary file 1 [file cimb-48-00103-s001.zip › Supplementary Files.pdf]

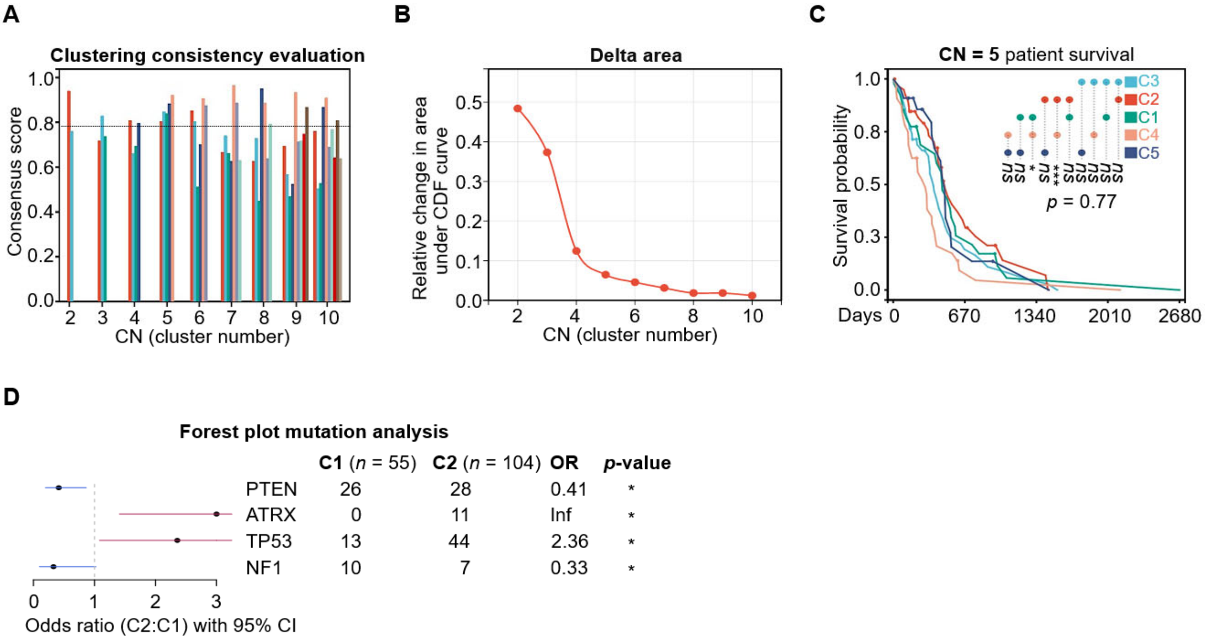

Supplementary Figure S1: Identification of Two GBM Prognostic Subtypes through Unsupervised Clustering.

(A) Clustering consistency evaluation of clustering results across different cluster numbers (CN), ranging from 2 to 10. The y-axis represents the consensus score, and the x-axis represents the cluster number. The dashed line marks the threshold for acceptable clustering consistency.

(B) Relative change in the area under the cumulative distribution function (CDF) curve for different cluster numbers comparing CN and CN-1.

(C) Kaplan-Meier survival curves for five cluster (CN = 5). Statistical significance is denoted as *p < 0.05, **p < 0.01, ***p < 0.001; ns indicates no significant difference.

(D) Forest plot of gene mutation anasysis of key genes (PTEN, ATRX, TP53, NF1) in two clusters. The plot displays the odds ratios (OR) with 95% confidence intervals (CI) for each gene, comparing mutation frequencies between the clusters. PTEN has an OR of 0.41, ATRX shows an infinite OR due to zero mutations in C1, TP53 has an OR of 2.36, and NF1 has an OR of 0.33. Statistical significance is denoted as *p < 0.05. Values less than 1 indicate more frequent mutations in C1.

A

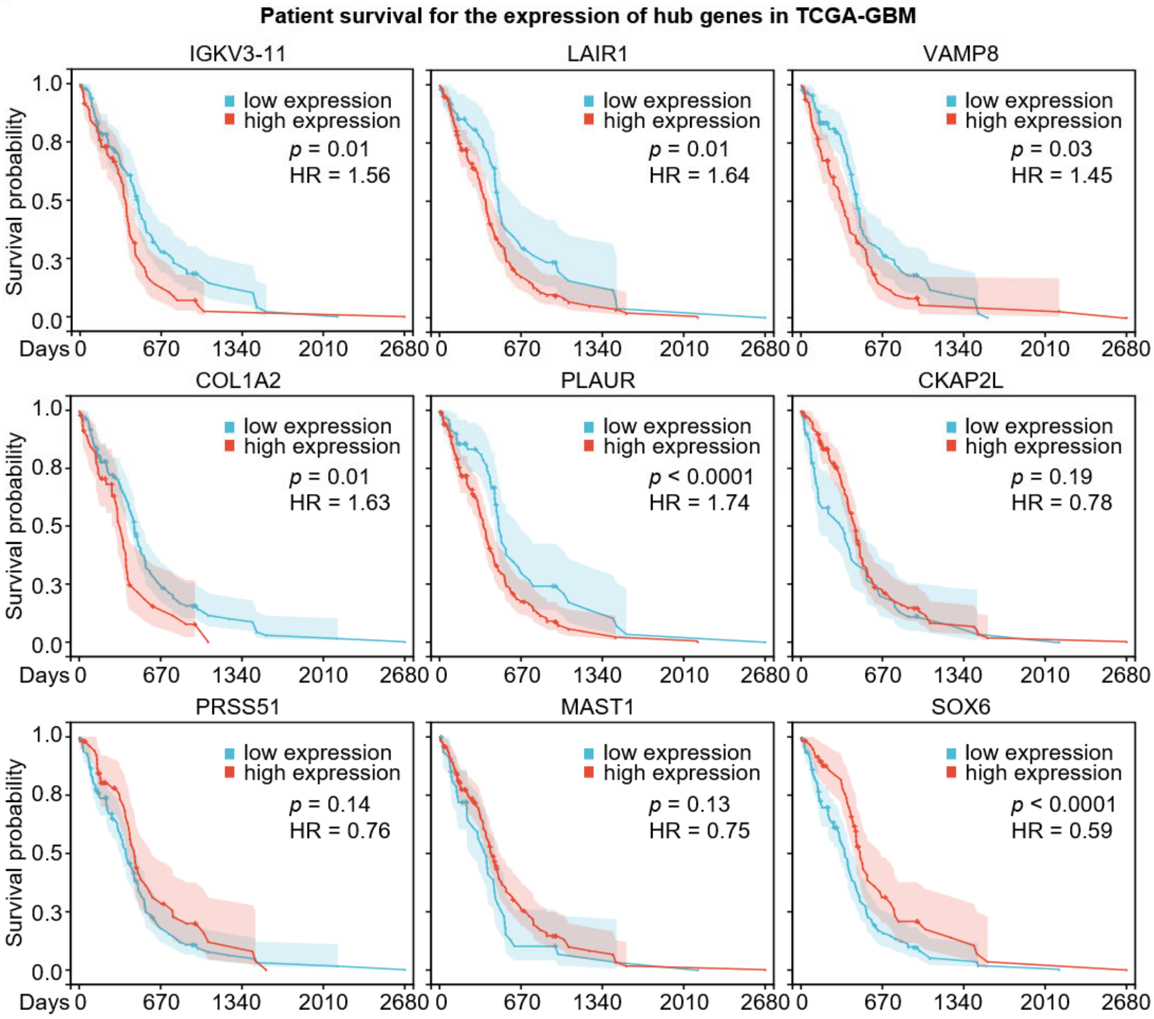

Supplementary Figure S2: Identification of featured biomarkers for C1/C2 subtypes via Weighted Correlation Network Analysis (WGCNA).

(A) Kaplan-Meier survival curves illustrating the prognostic significance of hub gene expression from the TCGA-GBM database. Survival probabilities were compared between patients stratified into low (blue) and high (red) expression groups for each of the identified hub genes, including IGKV3-11, LAIR1, VAMP8, COL1A2, PLAUR, CKAP2L, PRSS51, MAST1, and SOX6. Shaded areas represent the 95% confidence intervals.

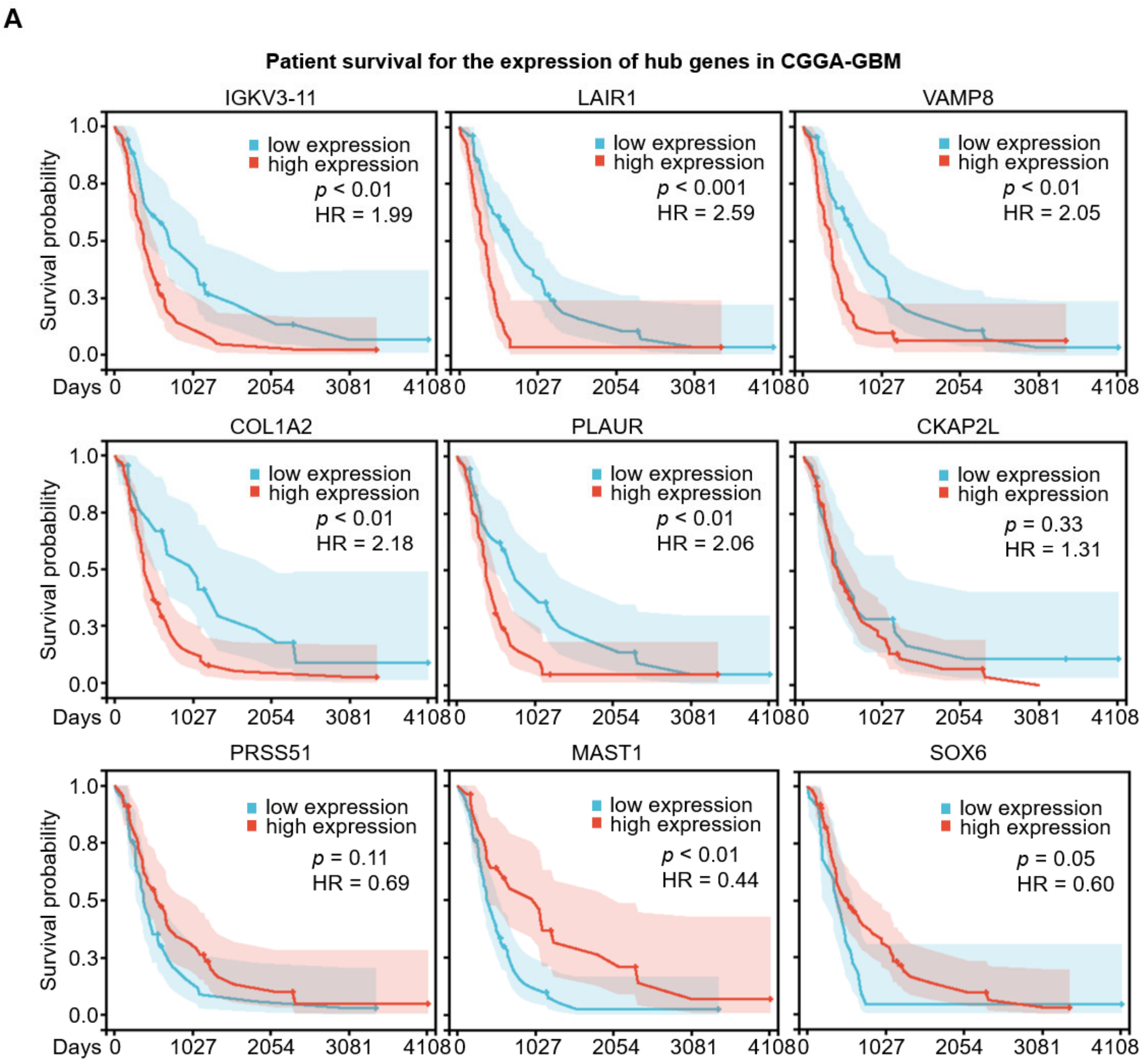

Supplementary Figure S3: Classification model utilizing machine learning on subtype-specific hub genes.

(A) Kaplan-Meier survival curves illustrating the prognostic significance of hub gene expression from the CGGA-GBM database. Survival probabilities were compared between patients stratified into low (blue) and high (red) expression groups for each of the identified hub genes, including IGKV3-11, LAIR1, VAMP8, COL1A2, PLAUR, CKAP2L, PRSS51, MAST1, and SOX6. Shaded areas represent the 95% confidence intervals.

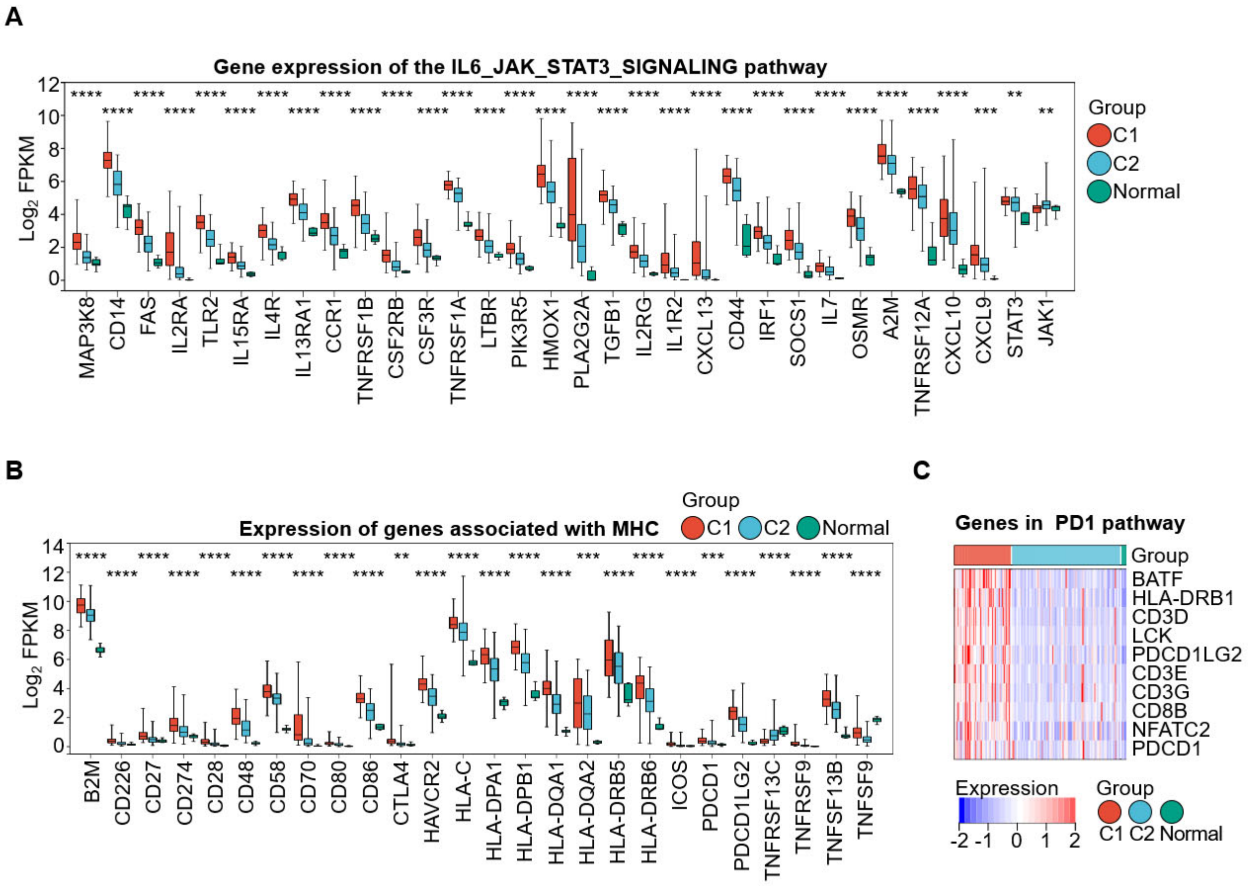

Supplementary Figure S4: C1 subtype: a "hot" tumor with rich immune cells in the tumor microenvironment.

(A) Gene expression of the IL6_JAK_STAT3_SIGNALING pathway in C1, C2, and normal samples in the TCGA-GBM dataset. Statistical significance denoted as *p < 0.05, **p < 0.01, ***p < 0.001, ****p < 0.0001.

(B) Analysis of gene expression related to major histocompatibility complex (MHC) in C1, C2, and normal samples in the TCGA-GBM dataset. Statistical significance denoted as *p < 0.05, **p < 0.01, ***p < 0.001, ****p < 0.0001.

(C) Gene expression heatmap of DEGs involved in the PD1 pathway.

A

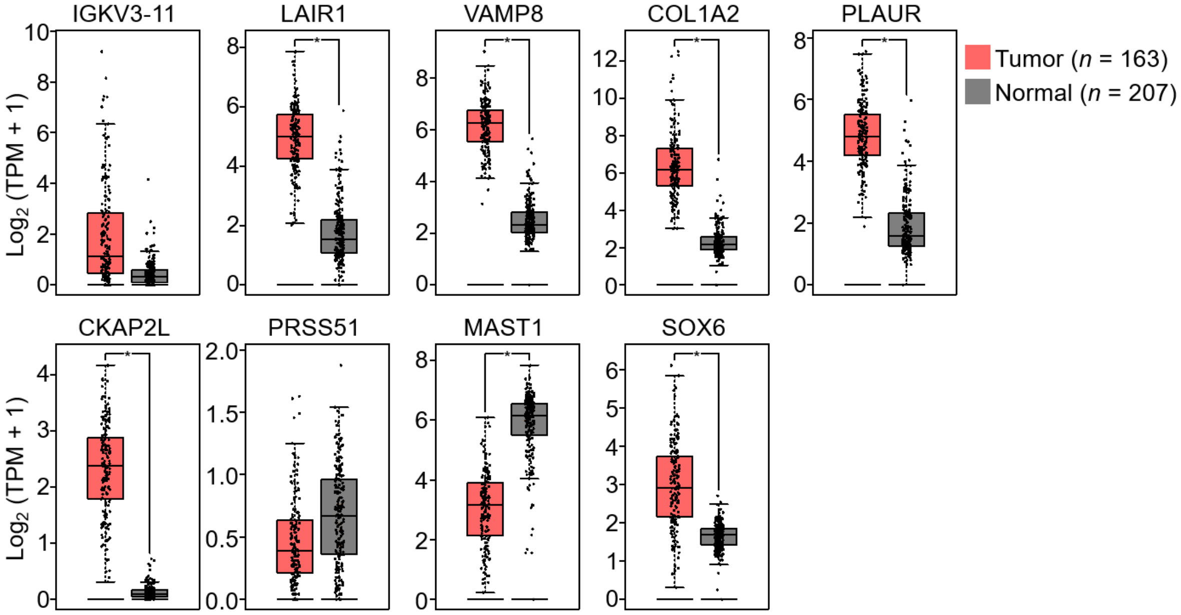

Supplementary Figure S5: External validation of the expression levels of the nine hub genes using the GEPIA2 database.

(A) Validation of the 9 hub genes expression in an independent cohort combining TCGA GBM samples and GTEx normal brain samples (n = 370). Statistical significance denoted as *p < 0.05.
